# Association between harmful alcohol use and periodontal status according to gender and smoking

**DOI:** 10.1186/1472-6831-14-73

**Published:** 2014-06-20

**Authors:** Hyang-Sun Kim, Ji-Hyun Son, Hee-Yong Yi, Hae-Kyoung Hong, Hyun-Jun Suh, Kwang-Hak Bae

**Affiliations:** 1 Department of Physiology, College of Medicine, Seoul National University, Seoul, Korea; 2 Korean Minjok Leadership Academy, Hoengseong-gun, Wonju, Gangwon-do, Korea; 3 Department of Preventive and Public Health Dentistry, School of Dentistry, Seoul National University, 28, Yeongeuon-dong, Jongno-gu, 110-749 Seoul, Republic of Korea; 4 Department of Dental Hygiene, Kyungdong University, Wonju, Gangwon-do, Korea; 5 Kyoung-ki Academy, Seoul, Korea; 6 Dental Research Institute, School of Dentistry, Seoul National University, Seoul, Korea

**Keywords:** Harmful Alcohol use, Periodontal status, Association, Gender, Smoking

## Abstract

**Background:**

the aim of this study is to assess the association of harmful alcohol use based on the alcohol use disorders identification test (AUDIT) score with periodontal status according to gender and smoking in a representative sample of Korean adults.

**Methods:**

This study analyzed 5,291 participants older than 19 years whose data of harmful alcohol use and periodontal status were available. Harmful alcohol use was defined by the WHO guidelines for the administration of AUDIT. The periodontal status was assessed by the Community Periodontal Index (CPI). Multivariate logistic regression analysis was performed with adjustment for socio-demographic variables, oral and general health behavior, oral health status and systemic conditions. All analyses considered a complex sampling design, and multivariate analysis was also performed in the subgroups.

**Results:**

Multivariate logistic regression analysis revealed a marginal association between harmful alcohol use and higher CPI in the total sample. The adjusted odds ratio (OR) of harmful alcohol use was 1.16 (0.97 to 1.38) for higher CPI. Higher CPI was significantly associated with harmful alcohol use in men (OR: 1.28; 95% CI: 1.03-1.60) and non-smokers (OR: 1.29; 95% CI: 1.06-1.57).

**Conclusion:**

Periodontal status is significantly associated with harmful alcohol use in men and non-smokers in a representative sample of Korean adults.

## Background

Periodontitis is a chronic and long-lasting low-grade inflammatory disease that leads to a break-down of the connective tissue and bone that anchors the teeth to the jaws [1,2]. It is a highly prevalent disease worldwide contributing to the global burden of chronic diseases and represents a major public health problem in many countries [3,4]. In Korea, the prevalence of periodontitis is 29.4% among adults [5]. Periodontitis is also related to systemic alterations such as atherosclerotic vascular disease and metabolic syndrome [6,7].

Long-term and excessive alcohol consumption can influence host defenses causing toxic damage, which is implicated in a wide variety of diseases, disorders, and injuries such as oro-pharyngeal cancer, liver cirrhosis, pancreatitis, hypertension, gastritis, diabetes, some forms of stroke, and mental disorders such as depression [8-10].

As chronic and even acute, moderate alcohol use can increase host susceptibility to infections caused by bacterial pathogens and impaired host defense after alcohol exposure appears to be linked to a combination of decreased inflammatory response, altered cytokine production, and abnormal reactive oxygen intermediate generation [11], it may be related to periodontitis independently of other potential confounders.

However, a recent systematic review reported that, among 14 studies, only 3 studies showed clear association, and 6 studies showed marginal or weak association of alcohol consumption or dependence with periodontitis, while the other 5 studies did not find significant association [10]. Moreover, there was much diversity in the way of measuring alcohol consumption, and most studies considered limited potential confounders or examined non-representative small samples [10,12,13]. Hence, further studies are needed to elucidate whether or not alcohol consumption is a risk factor for periodontitis.

The Alcohol use disorders identification test (AUDIT) was developed by the World Health Organization (WHO) and validated by lots of studies as a simple method of screening for excessive drinking as the cause of the presenting illness, which is the only screening test specifically designed for international use, and in comparison to other screening tests, the AUDIT has been found to perform equally well or at a higher degree of accuracy across a wide variety of criterion measures [14].

When investigating the periodontitis-associated factors, effect modification needs to be considered in an appropriate manner in epidemiological studies because multivariate models with a single estimate require homogeneity of the effect across different levels of extraneous variables such as gender and smoking [15].

However, there are few studies on the association between alcohol consumption based on AUDIT score and periodontitis in a nationally representative sample considering effect modification.

Therefore, the aim of this study is to assess the association of harmful alcohol use based on AUDIT score with periodontal status according to gender and smoking in a representative sample of Korean adults.

## Methods

### Study design and subject selection

The data included a subset of the Fourth Korea National Health and Nutrition Examination Survey (KNHANES) conducted in 2009 by the Korea Center for Disease Control and Prevention (KCDC). The sampling protocol for the KNHANES was designed to involve a complex, stratified, multistage, probability-cluster survey of a representative sample of the non-institutionalized civilian population in Korea. The survey was performed by the Korean Ministry of Health and Welfare. The target population of the survey was all non-institutionalized civilian Korean individuals aged 1 year or older. The survey employed stratified multistage probability sampling units based on geographic area, gender, and age, which were determined based on the household registries of the 2005 National Census Registry, the most recent 5-year national census in Korea. Using the 2005 census data, 200 primary sampling units (PSU) were selected across Korea. The final sample set for KNHANES included 4,600 households. Among 12,722 sampled individuals, the number of participants was 10,533. The response rate was 82.8%. A total of 7,893 individuals aged over 19 participated in KNHANES, but 5,291 of the participants, who received a periodontal examination, were examined for AUDIT. A detailed description of the sampling was described in the KNHANES report [5].

### Clinical variables

#### *Periodontal status*

The WHO community periodontal index (CPI) was used to assess periodontal status. Higher CPI was defined as a CPI greater than or equal to ‘code 3’, which indicates that at least one site had a > 3.5 mm (code 4 > 5.5 mm) – probing pocket depth. The index tooth numbers were 11, 16, 17, 26, 27, 31, 36, 37, 46 and 47.

A CPI probe that met the WHO guidelines was used [16]. The mouth was divided into sextants. An approximately 20 g probing force was used. In 2009 KNHANES, 27 trained dentists examined the periodontal status of the subjects. The inter-examiner mean of kappa value was 0.77 (0.53 to 0.94) [17].

#### *Harmful alcohol use*

The AUDIT was administered as a self-report questionnaire composed of 10 questions including frequency of drinking, typical quantity, frequency of heavy drinking, impaired control over drinking, increased salience of drinking, morning drinking, guilt after drinking, blackouts, alcohol-related injuries, and others concerned about drinking (see Additional file 1: Table S1). Each of the questions has a response with a score ranging from 0 to 4. The participants with a total AUDIT score of 8 or higher were assessed as harmful alcohol use according to the WHO guidelines for the administration of AUDIT [14].

#### *Covariates*

The socio-demographic variables included gender, age, household income, and educational level. Household income was the family income adjusting for the number of family members. The educational level was assessed by highest diploma.

The oral health behaviors included daily frequency of toothbrushing and use of dental floss or interdental brush. As general health behavior, current smoking status was included. According to the current smoking status, the participants were divided to 3 groups (Non-smokers: those who had never smoked or had smoked fewer than 100 cigarettes in their life, current smokers: those who were currently smoking and had smoked 100 cigarettes or more in their whole life, past smokers: those who had smoked in the past but they stopped smoking at that time).

The oral health status included the number of decayed permanent teeth (DT), which was examined and summarized according to WHO criteria [18]. The systemic conditions included diabetes and obesity.

### Statistical analysis

The individual weighted factors were used and the complex sampling design of the survey was considered to obtain the variances. Multivariate logistic regression analyses were applied to examine the relationships between harmful alcohol use and periodontal status. The odds ratios of harmful alcohol use for higher CPI were adjusted for above-mentioned covariates in logistic model. Because the interaction terms of periodontal status with gender and smoking was significant, subgroup analyses were performed to gain estimates stratified according to gender and smoking. Statistical analyses were performed using SPSS version 19.0 software (SPSS, Chicago, IL, USA).

## Results

The prevalence of higher CPI defined as a CPI code ≥ 3 was 30.9% (code 4 was 5.9%). The mean age of the participants was 39.13 (38.35 to 39.90) in the participants with lower CPI and 50.30 (49.24 to 51.36) in those with higher CPI. The mean number of DT of the participants was 0.79 (0.71 to 0.86) in the participants with lower CPI and 0.90 (0.79 to 1.01) in those with higher CPI. Table 1 list the characteristics of the study participants categorized by the periodontal status.

**Table 1:** Bivariate comparisons of the characteristics between the participants with lower and higher CPI

	**Lower CPI**		**Higher CPI**	
	**n**	%^* ^**(95% CI)**	**n**	%^* ^**(95% CI)**
Gender (n = 5291)				
Male	1444	63.2 (60.5-65.8)	965	36.8 (34.2-39.5)
Female	2176	77.6 (75.3-79.7)	706	22.4 (20.3-24.7)
Highest diploma (n = 5283)				
Primary school	666	52.0 (47.4-56.5)	497	48.0 (43.5-52.6)
Middle school	362	58.9 (53.8-63.9)	260	41.1 (36.1-46.2)
High school	1452	73.5 (70.7-76.2)	559	26.5 (23.8-29.3)
≥ University or college	1134	76.4 (73.7-78.9)	353	23.8 (21.1-26.3)
Household income^†^ (n = 5247)				
< 25%	581	63.1 (58.5-67.4)	366	36.9 (32.6-41.5)
25 - 50%	786	66.6 (63.4-69.7)	424	33.4 (30.3-36.6)
50 - 75%	1036	69.0 (65.6-72.2)	467	31.0 (27.8-34.4)
> 75%	1182	75.1 (72.4-77.6)	405	24.9 (22.4-27.6)
Diabetes (n = 5291)				
No	3450	71.2 (69.1-73.2)	1483	28.8 (26.8-30.9)
Yes	170	45.6 (39.4-52.0)	188	54.4 (48.0-60.6)
Obesity^‡^ (n = 5251)				
Underweight	179	77.8 (71.4-83.1)	55	22.2 (16.9-28.6)
Normal	2337	71.6 (69.3-73.8)	1024	28.4 (26.2-30.7)
Obesity	1068	64.5 (61.4-67.4)	588	35.5 (32.6-38.6)
Oral health behaviors				
Daily frequency of toothbrushing (n = 5291)				
Once or less	401	63.2 (58.7-67.6)	249	36.8 (32.4-41.3)
Twice	1381	66.8 (63.7-69.7)	738	33.2 (30.3.-36.3)
Three times or more	1838	73.7 (71.3-76.1)	684	26.3 (23.9-28.7)
Use of floss or interdental brush (n = 5291)				
No	2653	68.8 (66.4-71.2)	1316	31.2 (28.8-33.6)
Yes	967	72.7 (69.5-75.7)	355	27.3 (24.3-30.5)
General health behaviors				
Harmful alcohol use^§^ (n = 5291)				
No	2519	72.4 (70.0-74.6)	1048	27.6 (25.4-30.0)
Yes	1101	65.7 (62.8-68.5)	623	34.3 (31.5-37.2)
Present smoking status (n = 5282)				
Past smoker	662	62.0 (58.5-65.5)	450	38.0 (34.5-41.5)
Current smoker	776	61.8 (58.1-65.4)	515	38.2 (34.6-41.9)
Non-smoker	2213	78.0 (75.7-80.0)	719	22.0 (20.0-24.3)

^*^Weighted percent and 95% confidence interval.

^†^Household income: monthly average family equivalent income.

(=monthly average household income/√(the number of household members)).

^‡^Underweight (<18.5 kg/m2); Normal (18.5 to 24.9 kg/m2); obese (≥25 kg/m2).

^§^AUDIT score ≥ 8.

Table 2 showed the marginal association between harmful alcohol use and higher CPI in the multivariate logistic regression model in a total sample. Harmful alcohol use might be associated with higher CPI, but the strength of the association was marginal. The results of the subgroup analyses are also presented in Table 4. The association was different according to the strata of gender and smoking. While harmful alcohol use showed moderate association with higher CPI in males (OR: 1.28; 95% CI: 1.03-1.60) and non-smokers (OR: 1.29; 95% CI: 1.06-1.57), the significant association between harmful alcohol use and higher CPI was not found in females and current smokers.

**Table 2:** Adjusted odds ratios (OR) and 95% confidence intervals (CI) of harmful alcohol use (AUDIT score ≥ 8) for higher CPI in total sample and each subgroup

	**OR**	**95% CI**
Total	1.158	0.969-1.383
Gender		
Male	1.284	1.028-1.604
Female	0.803	0.565-1.139
Current smoker		
No	1.291	1.058-1.575
Yes	1.003	0.717-1.403

The multivariate logistic regression model was adjusted for socio-demographic variables (age, gender, household income, educational level), oral health behaviors (daily frequency of toothbrushing, use of floss or interdental brush), general health behaviors (present smoking status), oral health status (active caries) and general health status (diabetes mellitus and obesity).

In the subgroup, each effect modifier was excluded from its multivariate model.

## Discussion

In this study, an association was found between harmful alcohol use and periodontal status after adjusting for the socio-demographic variables, oral and general health behaviors, and oral health status, especially in men and non-smokers.

While alcohol consumption has been widely perceived as a risk factor of periodontitis due to the biological plausibility based on the relationship between alcohol use and impaired systemic conditions such as reduced resistance to infection and liver damage [19-21], several studies reported a negative correlation between alcohol consumption and periodontitis based on longitudinal and cross-sectional researches.

For example, Jansson [22] conducted a longitudinal investigation of 513 individuals from the County of Stockholm in 1970 and 1990. He did not find any significant association between alcohol consumption and periodontitis. Even though the subjects with higher alcohol consumption had calculus more frequently than those with lower alcohol consumption, alcohol consumption was not associated with periodontitis. Torrungruang et al. [23] performed a cross-sectional survey targeting 2,005 people aged 50 to 73 years in Thailand, and also reported that alcohol consumption had no significant effect on the severity of periodontal disease severity in the multivariate model.

This disparity might be due to differences in the potential confounders, assessment of alcohol consumption, criteria of periodontitis, and ethnical backgrounds. In addition, effect modification could be another reason for the variation. Most previous studies on the association between alcohol consumption and periodontitis did not consider effect modifiers. Ylöstalo et al. [15] reported that effect modification was not always treated in an appropriate manner in epidemiological studies although it was a basic concept in quantitative research. Based on the analysis of simulation data, they concluded that effect modification might explain the variation in the results of studies on the association between periodontitis and systemic disease. In this study, as we found significant interaction terms, subgroup analysis was performed with subgroups stratified by the levels of effect modifiers. The results of this study did not support the association between harmful alcohol use and periodontal status in the total sample. However, subgroup analysis showed that harmful alcohol use was significantly associated with higher CPI in men and non-smokers.

To the best of our knowledge, this is the first study to report gender and smoking as effect modifying factors of the association between alcohol consumption and periodontal status. Some studies reported that smoking and alcohol consumption are associated with increased risk of systemic diseases such as fatty liver disease and metabolic syndrome, especially when these two exposures occur together [24,25]. The result of this study is opposite to those previous results. However, considering that smoking is one of the strongest risk factors for periodontitis [26], it could be inferred that smoking may mask the effect of alcohol consumption on periodontal health in smokers.

Briasoulis et al. [27] found that the association between light to moderate alcohol intake and the risk of developing hypertension differed between women and men. They explained that differences in the pattern of drinking, beverage choices, and smoking habits may contribute to the observed sex differences because the health effects of drinking may depend on drinking pattern, and failure to differentiate episodic from regular drinkers may obscure the real associations. It is consistent with the gender difference found in this study.

Further studies will be needed to elucidate the association between alcohol consumption and periodontal status according to gender and smoking.

The difference in the assessment of alcohol consumption is also suggested to be one of the explanations for the variation in the results of the studies on the association between alcohol consumption and periodontal status. Amaral Cda et al. [10] reported that alcohol consumption was assessed by various unvalidated questionnaires with different criteria varied according to researchers in lots of studies. Therefore, this study used the AUDIT to determine harmful alcohol use.

The AUDIT was developed by the WHO and validated by lots of studies as a screening tool for excessive drinking [14]. Epidemiologic studies have used the AUDIT [28,29]. In order to evaluate the AUDIT against other common screening measures, Allen et al. [30] reviewed the studies in which the sensitivity and specificity of the AUDIT were contrasted with those of the alternative measures. They found that the AUDIT performed at a level at least comparable with and generally exceeding that of the alternate measures. However, there have been few studies on the association between periodontitis and alcohol consumption based on AUDIT [31].

This study had several limitations. The periodontal status was assessed by CPI. Although CPI is an easier way to assess the prevalence of periodontitis in a population survey and has been adopted as an index for periodontitis in epidemiologic studies on the association between systemic health and periodontal disease [32], the limitation of CPI should be deliberately considered since it can overestimate or underestimate the prevalence of periodontitis due to the use of representative teeth and pseudo pockets [33]. Therefore, the term of higher CPI was used instead of periodontitis to classify periodontal status. Another important limitation of this study is its cross-sectional design, which makes it impossible to determine the direction of the causal relationship of harmful alcohol use with periodontal status. As the association of subgroups found in this study was so weak, further studies will be needed to confirm the association between harmful alcohol use and periodontal status in men and non-smokers.

Nevertheless, this is the first epidemiologic study to report an effect modification of the association between harmful alcohol use and periodontal status by gender and smoking in a nationally representative sample of adults.

## Conclusion

Periodontal status is significantly associated with harmful alcohol use defined using the AUDIT in men and non-smokers. The underlying mechanisms showing an effect modification of the association between harmful alcohol use and periodontal status by gender and smoking remain to be determined through prospective cohort studies.

### Human subjects approval statement

The study was conducted in compliance with the principles of the Helsinki Declaration. Ethical clearance of the study was approved by the Institutional Review Board (IRB) of Korea Centers for Disease Control and Prevention (IRB number. 2009-01CON-03-2C).

## Competing interests

The authors declare that they have no competing interests.

## Authors’ contributions

BK designed the study and, YH and HH performed the statistical analysis. KH, SJ, and SH interpreted the findings and drafted the manuscript. BK read and approved the final manuscript. All Authors read and approve the final manuscript.

## Pre-publication history

The pre-publication history for this paper can be accessed here:

http://www.biomedcentral.com/1472-6831/14/73/prepub

## Supplementary Material

Additional file 1: Table S1 The Alcohol Use Disorders Identification Test [14]. Click here for file
