# Supplementary material for: Association between harmful alcohol use and periodontal status according to gender and smoking
Source: BMC Oral Health. 2014 Jun 20;14:73. doi: 10.1186/1472-6831-14-73 (PMC4114163; doi:10.1186/1472-6831-14-73)
Supplement: Additional file 1: Table S1 — The Alcohol Use Disorders Identification Test [14]. [file 1472-6831-14-73-S1.doc]

Additional file 1: Table S1. The Alcohol Use Disorders Identification Test [14]

| 1. How often do you have a drink containing alcohol?  (0) Never [Skip to Qs 9-10]  (1) Monthly or less  (2) 2 to 4 times a month  (3) 2 to 3 times a week  (4) 4 or more times a week | 6. How often during the last year have you needed  a first drink in the morning to get yourself going after a heavy drinking session?  (0) Never  (1) Less than monthly  (2) Monthly  (3) Weekly  (4) Daily or almost daily |
| --- | --- |
| 2. How many drinks containing alcohol do you have on a typical day when you are drinking?  (0) 1 or 2  (1) 3 or 4  (2) 5 or 6  (3) 7, 8, or 9  (4) 10 or more | 7. How often during the last year have you had a feeling of guilt or remorse after drinking?  (0) Never  (1) Less than monthly  (2) Monthly  (3) Weekly  (4) Daily or almost daily |
| 3. How often do you have six or more drinks on one occasion?  (0) Never  (1) Less than monthly  (2) Monthly  (3) Weekly  (4) Daily or almost daily  Skip to Questions 9 and 10 if Total Score  for Questions 2 and 3 = 0 | 8. How often during the last year have you been unable to remember what happened the night before because you had been drinking?  (0) Never  (1) Less than monthly  (2) Monthly  (3) Weekly  (4) Daily or almost daily |
| 4. How often during the last year have you found that you were not able to stop drinking once you had started?  (0) Never  (1) Less than monthly  (2) Monthly  (3) Weekly  (4) Daily or almost daily | 9. Have you or someone else been injured as a result of your drinking?  (0) No  (2) Yes, but not in the last year  (4) Yes, during the last year |
| 5. How often during the last year have you failed to do what was normally expected from you because of drinking?  (0) Never  (1) Less than monthly  (2) Monthly  (3) Weekly  (4) Daily or almost daily | 10. Has a relative or friend or a doctor or another health worker been concerned about your drinking or suggested you cut down?  (0) No  (2) Yes, but not in the last year  (4) Yes, during the last year |
